# Application effect of parental accompanying during anesthesia induction and recovery period nursing in endoscopic plasma-assisted tonsillectomy combined with adenoidectomy

**DOI:** 10.3389/fmed.2026.1771384

**Published:** 2026-04-24

**Authors:** Yu Yang, Chuan He, Jiayin Qin, Liqiu Wu

**Affiliations:** 1 Department of Operating Room, The People’s Hospital of Bozhou, Bozhou, Anhui, China; 2 Department of Anesthesiology, The People’s Hospital of Bozhou, Bozhou, Anhui, China

**Keywords:** anesthesia induction, children, delirium, parents, tonsillectomy and adenoidectomy

## Abstract

**Aim:**

To assess the application effect of parental accompanying during anesthesia induction and recovery period nursing in endoscopic plasma-assisted tonsillectomy combined with adenoidectomy.

**Methods:**

A total of 116 children admitted to our hospital between January 2021 to February 2024 who were diagnosed with adenotonsillar hypertrophy and scheduled to undergo endoscopic plasma-assisted tonsillectomy combined with adenoidectomy were enrolled in this study. Eligible children were identified from patients already posted for surgery during the study period and were assigned to the control group or the observation group, with 58 cases in each group. In the control group, anesthesia induction and recovery were managed by nurses alone, whereas in the observation group, parental presence was provided during anesthesia induction and nursing care during anesthesia recovery. The anesthesia resuscitation time, anxiety levels of children and parents, degree of cooperation during anesthesia induction, degree of delirium, degree of pain, and nursing satisfaction were compared between the two groups.

**Results:**

Compared with the control group, the observation group had a shorter eye-opening time and a shorter stay in the anesthesia resuscitation room (*P* < 0.01), lower mYPAS and S-TAI scores 1 day after surgery (*P* < 0.05), a lower ICC score (*P* < 0.01), lower PAED scores at T2, T3, and T4 (*P* < 0.01), lower FLACC scores at T2, T3, T4, and T5 (*P* < 0.01), and a higher nursing satisfaction rate (*P* < 0.05).

**Conclusion:**

Parental accompanying during anesthesia induction and recovery period nursing was associated with lower anxiety-related scores, better induction compliance, less emergence delirium and pain-related behavioral distress, and improved recovery-room outcomes in children undergoing endoscopic plasma-assisted tonsillectomy combined with adenoidectomy. However, these findings should be interpreted cautiously in light of the bundled nature of the intervention and the methodological limitations of the study.

## Introduction

Tonsillar and adenoidal hypertrophy is one of the most common upper airway disorders in children and is a major indication for tonsillectomy with or without adenoidectomy, which is frequently performed under general anesthesia in pediatric practice (1, 2). Perioperative anxiety is common in pediatric patients and may be triggered by separation from parents, unfamiliar surroundings, fear of medical procedures, and developmental immaturity, all of which can negatively affect cooperation during induction of anesthesia and the overall perioperative experience (3, 4). In addition to preoperative anxiety, emergence delirium represents another important perioperative concern in children recovering from general anesthesia (5, 6). Emergence delirium may present as agitation, crying, inconsolability, disorientation, or non-cooperation, and it can interfere with monitoring, increase nursing burden, and adversely affect the quality of early postoperative recovery (5, 7). This issue is particularly relevant in pediatric tonsillectomy and adenoidectomy, as prospective evidence in children undergoing these procedures has shown that postoperative emergence delirium is not uncommon and is closely associated with perioperative behavioral and pain-related factor (8). Perioperative care must prioritize not only surgical safety but also the child’s psychological and behavioral responses during both anesthesia induction and postoperative recovery.

Parental accompaniment has attracted increasing attention as a non-pharmacological intervention in pediatric perioperative care, especially during anesthesia induction, because the presence of a familiar caregiver may enhance the child’s sense of security and help reduce separation-related distress (3, 9). Recent evidence suggests that parental presence during induction may alleviate perioperative anxiety to some extent, although the magnitude of its benefit may vary across settings and comparators (3). Moreover, parental presence during the post-anesthesia care unit period has also been investigated, and a recent systematic review and meta-analysis reported that parental presence in the post-anesthesia care unit (PACU) can reduce emergence delirium scores in pediatric patients after general anesthesia (7). These findings indicate that parental accompaniment may have value across both the induction and recovery phases, yet evidence focusing on a continuous parent-accompanied perioperative nursing model in children undergoing tonsillectomy combined with adenoidectomy remains limited.

Herein, the aim of this study was to evaluate the effect of parental accompanying during anesthesia induction and recovery period nursing in children undergoing endoscopic plasma-assisted tonsillectomy combined with adenoidectomy. The primary objective was to determine whether parental accompaniment could reduce perioperative anxiety in children undergoing endoscopic plasma-assisted tonsillectomy combined with adenoidectomy. The secondary objectives were to assess its effects on induction compliance, emergence delirium, postoperative pain, anesthesia resuscitation time, and parental satisfaction.

## Data and methods

### Study patients

A total of 116 children admitted to our hospital between January 2021 and February 2024 who were diagnosed with adenotonsillar hypertrophy and scheduled to undergo endoscopic plasma-assisted tonsillectomy combined with adenoidectomy (adenotonsillectomy) were enrolled in this study. Eligible patients were identified from the surgical schedule of the Department of Otorhinolaryngology during the study period. The children were then assigned to the control group and the observation group, with 58 cases in each group. A CONSORT flow diagram illustrating participant enrollment, allocation, follow-up, and analysis has been added as Supplementary Figure 1.

Inclusion criteria: (1) children diagnosed with adenoid hypertrophy and tonsillar hypertrophy (adenotonsillar hypertrophy) based on polysomnography and nasal endoscopy; (2) children who had already been evaluated by the otolaryngology team and were scheduled for endoscopic plasma-assisted adenotonsillectomy; (3) children presenting with symptoms such as snoring during sleep, mouth breathing, or sleep-disordered breathing; and (4) children whose parents or legal guardians agreed to participate and signed the informed consent form. Exclusion criteria: (1) congenital anatomical abnormalities of the upper airway or throat; (2) severe organ dysfunction; (3) contraindications to surgery or general anesthesia; (4) hematological disorders; (5) psychiatric or neurological disorders affecting perioperative assessment; and (6) withdrawal from the study by the child’s parents or legal guardians.

### Randomization and blinding

This study was a randomized, parallel-group, controlled trial. Eligible children were randomly assigned to either the control group or the observation group in a 1:1 ratio using a simple randomization method. The randomization sequence was generated by a biostatistician not involved in patient enrollment or outcome assessment using SPSS software (version 26.0). The allocation sequence was concealed in sequentially numbered, opaque, sealed envelopes. After a child was confirmed to meet the inclusion criteria and written informed consent was obtained from the parents, a dedicated operating room nurse (who was not involved in outcome assessment) opened the next envelope in sequence to determine group assignment.

Due to the nature of the intervention (parental accompaniment during anesthesia induction and recovery), it was not possible to blind the children, parents, or the clinical staff delivering the intervention. However, to minimize detection bias, outcome assessors were blinded to group allocation. Specifically, (1) the research nurse who assessed all outcomes was different from the nurse who performed randomization and did not have access to the allocation list; (2) data on anesthesia resuscitation time were extracted from the medical records by a research assistant who was unaware of group assignment; and (3) statistical analysis was performed by a biostatistician who was blinded to group allocation, with the dataset coded as Group A and Group B. The code was broken only after all analyses were completed.

### Anesthesia induction

Children fasted according to institutional preoperative fasting guidelines before anesthesia. After arrival in the operating room, peripheral intravenous access was established for intravenous infusion, oxygen was administered via face mask, and heart rate, mean arterial pressure, and blood oxygen saturation were monitored. Maintenance intravenous fluid (normal saline) was infused at a rate of 6–8 mL/kg/h for 15 min before anesthesia induction to maintain venous access patency and provide basic fluid replacement after preoperative fasting. The induction agents were sufentanil 0.3–0.5 μg/kg, propofol 2–3 mg/kg, and rocuronium 0.6 mg/kg or cisatracurium 0.2 mg/kg. The final dose of sufentanil was determined according to body weight and titrated to achieve adequate depth of anesthesia without significant hemodynamic fluctuation. Anesthesia was maintained with sevoflurane in a mixture of oxygen and air (oxygen concentration 50%, total gas flow 2 L/min), titrated to maintain an end-tidal sevoflurane concentration of 1%–2% based on hemodynamic response and depth of anesthesia monitoring. Depth of anesthesia was monitored clinically based on heart rate, mean arterial pressure, and end-tidal sevoflurane concentration. Remifentanil was continuously infused at 0.1–0.3 μg/kg/min and propofol at 3–6 mg/kg/h, with doses titrated according to hemodynamic response and anesthetic requirements. Sevoflurane administration was discontinued 15 min before the end of surgery. Tracheal extubation was performed after the child regained spontaneous breathing, recovered airway protective reflexes, and met routine extubation criteria. If no intraoperative abnormalities were observed, the child was transferred to the PACU.

### Surgical method

All surgeries were performed under general anesthesia using a low-temperature plasma ablation system (Coblator II, ArthroCare, United States) with endoscopic guidance. Tonsillectomy was performed using a plasma knife head to dissect along the tonsillar capsule, followed by adenoidectomy under a 70° nasal endoscope. Hemostasis was confirmed before completion of the procedure.

### Nursing methods

The parents of children in two groups were taught by Operating room nurse about surgery and anesthesia on the day before surgery. The main education content included the basic process of surgery, the basic process of anesthesia and precautions. Operating room nurse played a 10 min promotional video to children and parents, so that parents could more intuitively understand the general process of anesthesia induction and recovery, and parents could have a better understanding of the operating room environment and the principle of aseptic disinfection through videos, so as to obtain parents’ understanding and cooperation. Operating room nurse explained to parents how to effectively soothe the child, such as cuddling, telling stories, telling jokes, and singing children’s songs. For older children, they were encouraged and guided, such as praising the courage of the children, and praising their spirit of cooperation with medical staff. Finally, the operating room nurse led the parents of the observation group to learn the principle of asepsis in the operating room, the wearing of surgical gowns and the method of rapid hand disinfection. In the observation group, one accompanying parent (either the father or the mother) was selected by the family, usually based on who was the child’s primary caregiver. The selected parent was required to be willing to participate, able to understand and follow staff instructions, and able to comply with operating-room requirements. On the day of surgery, children in the observation group were accompanied by one parent together with the operating room nurse to the anesthesia preparation room before anesthesia induction. Parents were allowed to remain with the child during the pre-induction period only; however, they were not present during anesthetic drug administration, loss of consciousness, or tracheal extubation. Children in the control group were accompanied only by the operating room nurse to the anesthesia preparation room and did not receive parent-present accompaniment during either the pre-induction or recovery phase.

After surgery, children in the control group were transferred to the anesthesia resuscitation room/PACU, where routine nursing care was provided by nurses, including vital-sign monitoring, warming measures, and verbal comfort and encouragement after awakening. Tracheal extubation was performed by medical staff once extubation criteria were met. No parent-present intervention was implemented in the control group during either the pre-induction or recovery phase. Apart from the routine preoperative education and simple comforting guidance provided on the day before surgery, no additional parent-accompanied non-pharmacological intervention was used in the control group.

After surgery, children in the observation group were transferred to the anesthesia resuscitation room/PACU, where vital signs were routinely monitored. Under the guidance of recovery-room staff, parents entered the designated area after putting on isolation clothing, a surgical cap, and special operating-room shoes. Parents were present during the early postoperative recovery period in the PACU; however, they were not present at the time of tracheal extubation. The recovery-room doctor and nurses explained the child’s postoperative condition and instructed the parents on how to cooperate during the recovery phase. When the child had not fully recovered and exhibited pain or restlessness, parents were guided to call the child’s name approximately every 20 s, hold the child’s hand, and gently touch the child’s forehead or face. Nurses also used distraction measures, including the child’s favorite songs or cartoons, favorite toys, praise, and encouragement, while closely monitoring vital signs until the child fully recovered.

### Observation indicators

The primary outcome of this study was perioperative anxiety. Secondary outcomes included induction compliance, emergence delirium, postoperative pain, anesthesia resuscitation time, and parental satisfaction.

(1) Anesthesia resuscitation time including the recovery time of spontaneous respiration, eye opening time of exhalation and stay time of anesthesia resuscitation room were compared between two groups.

(2) The Modified Yale Perioperative Anxiety Scale (mYPAS) (10) and State-Trait Anxiety Scale (S-TAI) (11), were used to assess anxiety before surgery and again on postoperative day 1. This time point was selected to reflect the overall perioperative psychological response, including the early postoperative recovery period, rather than the immediate anxiety state at the exact time of anesthesia induction. mYPAS contained five dimensions, namely, dependence on parents, arousal state, emotional expression, language and activity. The scale scores ranged from 23.3 points to 100 points, and the higher the score, the more anxiety was indicated. The S-TAI scale consisted of Trait Anxiety Scale (T-AT) and State Anxiety Scale (S-AT), both of which contained 20 items (40 items in total), and were evaluated by the scale of 1–4, with 1 point for no anxiety, two points for some anxiety, three points for moderate anxiety, and four points for significant anxiety. The score ranged from 40 to 140 points.

(3) Using Induction Compliance Checklist (ICC) (12), was used to evaluate the child’s cooperation during anesthesia induction, and the assessment was performed at the time of anesthesia induction. The scale contained 11 items, and each item was scored from 0 to 10 points. A total of 0 points indicated smooth induction and 10 points indicated uncooperative induction.

(4) Using Pediatric Anesthesia Emergence Delirium (PAED) scale (13), the degree of delirium in the child was assessed. This scale five items, each scored on a 0–4 Likert scale, giving a total score was 0∼20. The higher the total score was, the more serious the degree of delirium was. An overall score of ≥12 was defined as delirium. The children in both groups were evaluated for delirium every 10 min after entering the resuscitation room. The starting point (T_1_) was 5 min before entering the resuscitation room, the first 10 min after entering the resuscitation room was T_2_, the second 10 min was T_3_, the third 10 min was T_4_, and the exit room was T_5_.

(5) The Face, Legs, Activity, Cry, Consolability (FLACC) scale (14) as used to assess postoperative pain in children. The scale includes five dimensions with a total score ranging from 0 to 10, with higher scores indicating more severe pain. Pain was assessed at predefined time points during the post-anesthesia care unit stay, corresponding to T2–T5, consistent with the PAED assessment time points.

(6) Nursing satisfaction was assessed using a self-designed “Medical and Nursing Service Satisfaction Questionnaire.” The questionnaire was developed based on a review of relevant literature and clinical experience in perioperative pediatric nursing, and its content validity was reviewed by three senior nursing experts (two from the operating room and one from the anesthesia recovery unit) to ensure relevance and comprehensiveness. The questionnaire consists of 10 items covering five domains: communication with medical staff, explanation of procedures, pain management, emotional support, and overall satisfaction. Each item is scored on a 10-point Likert scale (1 = strongly dissatisfied, 10 = strongly satisfied), with a total score ranging from 10 to 100. Based on the total score, satisfaction was categorized as very satisfied (≥95), basically satisfied (90–94), or dissatisfied (<90). The total satisfaction rate was calculated as (very satisfied cases + basically satisfied cases)/total cases × 100%. Internal consistency of the questionnaire in this study was acceptable, with a Cronbach’s α coefficient of 0.87.

All scale-based outcomes, including mYPAS, S-TAI, ICC, PAED, and FLACC, were assessed by the same trained research nurse, who was different from the nurse responsible for randomization and intervention delivery and had no access to the allocation list. Anesthesia resuscitation time was extracted from the medical record by a separate research assistant who was blinded to group assignment. Family satisfaction questionnaires were completed independently by parents and collected by study staff not involved in intervention delivery. These procedures were used to minimize detection bias and maintain observer neutrality.

### Statistical analysis

SPSS 26.0 software was used for statistical analysis of data. Normality of continuous data was assessed using the Shapiro-Wilk test. All outcome variables (mYPAS, S-TAI, ICC, PAED, FLACC) were found to be approximately normally distributed (*P* > 0.05 for all groups at all time points). To further verify the robustness of the results, sensitivity analyses were performed using non-parametric tests (Mann-Whitney U test) for comparisons between groups; the results were consistent with those from parametric tests (all *P* < 0.05). For comparisons between two groups at a single time point, independent *t*-tests were used. For repeated measures data (PAED and FLACC scores assessed at multiple time points), a two-way repeated-measures analysis of variance (ANOVA) was performed, with group (control vs. observation) as the between-subjects factor and time (T_1_–T_5_) as the within-subjects factor. The Greenhouse-Geisser correction was applied when the assumption of sphericity was violated. *Post hoc* comparisons were performed using Bonferroni correction to adjust for multiple comparisons. Effect sizes for between-group comparisons were calculated using Cohen’s d, with values of 0.2, 0.5, and 0.8 interpreted as small, medium, and large effects, respectively. For repeated-measures ANOVA, partial η^2^ was reported as a measure of effect size. Additionally, 95% confidence intervals (CIs) were calculated for all key outcome measures to facilitate interpretation of clinical significance. *P* < 0.05 indicated that the difference was statistically significant. No formal *a priori* sample size calculation was performed before the study. The sample size was determined by the number of eligible children who underwent endoscopic plasma-assisted tonsillectomy combined with adenoidectomy during the study period and met the inclusion criteria.

## Results

### Baseline characteristics of the two groups

A total of 116 children were included in the study, with 58 cases in the control group and 58 cases in the observation group. The baseline demographic characteristics of the two groups are shown in Table 1. There were no significant differences between the two groups in age, sex, body weight, or ASA physical status (all *P* > 0.05).

**TABLE 1:** Baseline characteristics of children in both groups.

Characteristic	Control group (*n* = 58)	Observation group (*n* = 58)	Statistic (t/χ ^2^)	*P*-value
Age (years, mean ± SD)	6.29 ± 1.95	6.90 ± 1.78	1.741	0.084
Sex, *n* (%)	–	–	1.848	0.174
Male	41 (70.7)	34 (58.6)	–	–
Female	17 (29.3)	24 (41.4)	–	–
Body weight (kg, mean ± SD)	23.8 ± 5.2	24.5 ± 5.6	0.701	0.485
ASA physical status, *n* (%)	–	–	0.000	1.000
I	45 (77.6)	46 (79.3)	–	–
II	13 (22.4)	12 (20.7)	–	–

ASA, American Society of Anesthesiologists. *P*-values were calculated using independent *t*-test for continuous variables and chi-square test for categorical variables.

### Anesthesia resuscitation time between the two groups

**FIGURE 1:**
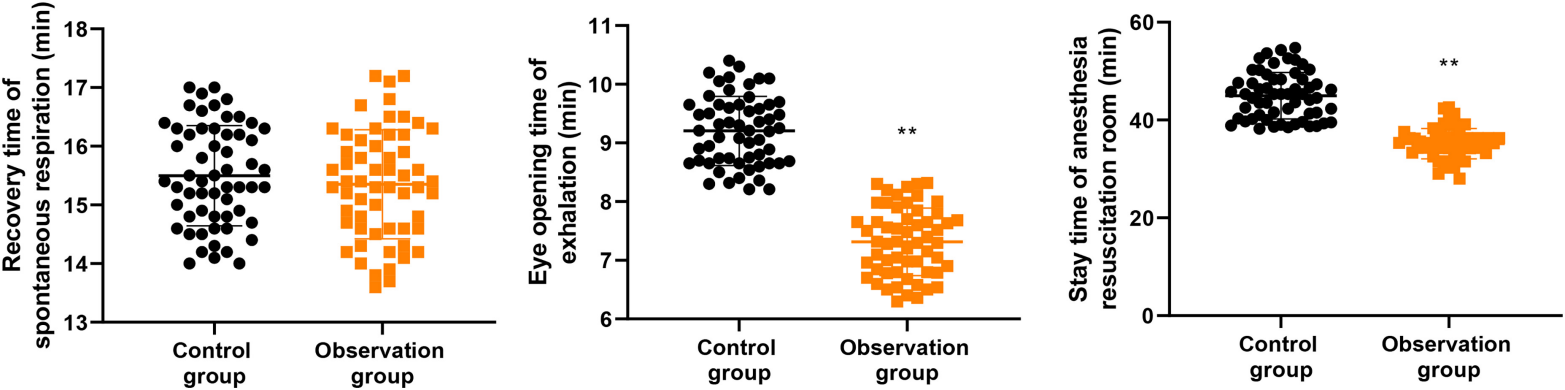
Anesthesia resuscitation time between two groups. **P < 0.01.

As Figure 1 displayed, relative to the control group, the observation group presented shorter eye-opening time of exhalation (mean difference = 1.91 min, 95% CI [1.68, 2.14], Cohen’s *d* = 2.52, *P* < 0.001) and stay time of anesthesia resuscitation room (mean difference = 10.69 min, 95% CI [8.90, 12.48], Cohen’s *d* = 1.97, *P* < 0.001). However, the recovery time of spontaneous respiration showed no significant difference between the two groups (mean difference = 0.03 min, 95% CI [−0.26, 0.32], Cohen’s *d* = 0.04, *P* = 0.839) (Table 2).

**TABLE 2:** Key outcome measures and effect sizes.

Outcome	Time point/indicator	Control group (*n* = 58)	Observation group (*n* = 58)	Mean difference	95% CI	Cohen’s d/partial η ^2^	*P*-value
Anesthesia resuscitation time							
Recovery time of spontaneous respiration (min)	–	15.44 ± 0.92	15.41 ± 0.92	0.03	[−0.26, 0.32]	0.04	0.839
Eye opening time of exhalation (min)	–	9.15 ± 0.62	7.24 ± 0.58	1.91	[1.68, 2.14]	2.52	<0.001
Stay time of anesthesia resuscitation room (min)	–	45.27 ± 5.03	34.58 ± 4.72	10.69	[8.90, 12.48]	1.97	<0.001
Anxiety (mYPAS)							
	Before surgery	28.65 ± 2.87	29.15 ± 3.05	0.50	[−0.56, 1.56]	0.16	0.353
	1 day after surgery	58.68 ± 6.02	45.68 ± 4.62	13.00	[10.97, 15.03]	2.46	<0.001
Anxiety (S-TAI)							
	Before surgery	39.76 ± 4.02	40.15 ± 4.06	0.39	[−1.08, 1.86]	0.10	0.602
	1 day after surgery	59.72 ± 6.15	49.25 ± 5.13	10.47	[8.42, 12.52]	1.86	<0.001
Induction compliance (ICC)							
	ICC score	5.34 ± 1.57	3.16 ± 1.60	2.18	[1.72, 2.64]	1.36	<0.001
Emergence delirium (PAED)							
	T_1_ (baseline)	14.65 ± 1.48	14.66 ± 1.47	−0.01	[−0.55, 0.53]	0.01	0.971
	T_2_	13.65 ± 1.37	9.72 ± 0.98	3.93	[3.49, 4.37]	3.28	<0.001
	T_3_	10.52 ± 1.05	8.54 ± 0.85	1.98	[1.62, 2.34]	2.08	<0.001
	T_4_	9.27 ± 0.93	5.36 ± 0.53	3.91	[3.63, 4.19]	5.09	<0.001
	T_5_	5.12 ± 0.52	5.13 ± 0.51	−0.01	[−0.20, 0.18]	0.02	0.917
Pain (FLACC)							
	T_1_ (baseline)	7.69 ± 0.78	7.72 ± 0.75	−0.03	[−0.31, 0.25]	0.04	0.832
	T_2_	7.23 ± 0.73	4.75 ± 0.48	2.48	[2.25, 2.71]	3.98	<0.001
	T_3_	6.17 ± 0.62	3.25 ± 0.33	2.92	[2.74, 3.10]	5.85	<0.001
	T_4_	5.32 ± 0.53	3.12 ± 0.31	2.20	[2.04, 2.36]	5.07	<0.001
	T_5_	4.89 ± 0.49	2.73 ± 0.28	2.16	[2.01, 2.31]	5.37	<0.001
Repeated-measures ANOVA							
PAED – group effect	–	–	–	–	–	Partial η^2^ = 0.20	<0.001
PAED – time effect	–	–	–	–	–	Partial η^2^ = 0.12	<0.001
PAED – group × time	–	–	–	–	–	Partial η^2^ = 0.08	<0.001
FLACC – group effect	–	–	–	–	–	Partial η^2^ = 0.22	<0.001
FLACC – time effect	–	–	–	–	–	partial η^2^ = 0.16	< 0.001
FLACC – group × time	–	–	–	–	–	Partial η^2^ = 0.09	<0.001
Within-group change (pre vs. post)							
mYPAS – control group	–	–	–	30.03	[28.14, 31.92]	5.73	<0.001
mYPAS – observation group	–	–	–	16.53	[15.06, 18.00]	3.89	<0.001
S-TAI – control group	–	–	–	19.96	[17.96, 21.96]	3.76	<0.001
S-TAI – observation group	–	–	–	9.10	[7.42, 10.78]	1.99	<0.001

Data are presented as mean ± SD unless otherwise indicated. For single time point comparisons, mean difference and 95% CI are shown. For repeated-measures ANOVA, partial η^2^ is reported as effect size. CI, confidence interval.

### Anxiety degree of children and parents at different times between the two groups

**FIGURE 2:**
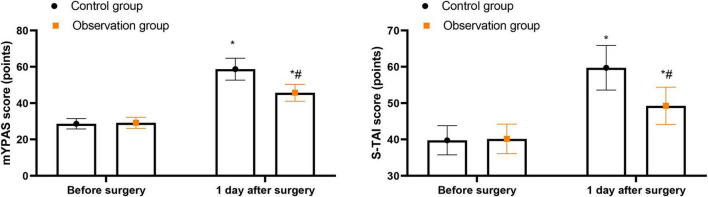
Anxiety degree of children and parents at different times between two groups. *P < 0.05 vs. before surgery; ^#^*P* < 0.05 vs. control group.

As Figure 2 displayed, mYPAS scores showed no significant difference between the two groups before surgery (mean difference = 0.50, 95% CI [−0.56, 1.56], Cohen’s *d* = 0.16, *P* = 0.353). On postoperative day 1, mYPAS scores were significantly elevated in both groups compared with preoperative values (control group: mean difference = 30.03, 95% CI [28.14, 31.92], Cohen’s *d* = 5.73, *P* < 0.001; observation group: mean difference = 16.53, 95% CI [15.06, 18.00], Cohen’s *d* = 3.89, *P* < 0.001). Importantly, the observation group had significantly lower mYPAS scores than the control group on postoperative day 1 (mean difference = 13.00, 95% CI [10.97, 15.03], Cohen’s *d* = 2.46, *P* < 0.001) (Table 2). Similarly, S-TAI scores showed no significant difference between the two groups before surgery (mean difference = 0.39, 95% CI [−1.08, 1.86], Cohen’s *d* = 0.10, *P* = 0.602). On postoperative day 1, S-TAI scores were significantly elevated in both groups compared with preoperative values (control group: mean difference = 19.96, 95% CI [17.96, 21.96], Cohen’s *d* = 3.76, *P* < 0.001; observation group: mean difference = 9.10, 95% CI [7.42, 10.78], Cohen’s *d* = 1.99, *P* < 0.001). The observation group had significantly lower S-TAI scores than the control group on postoperative day 1 (mean difference = 10.47, 95% CI [8.42, 12.52], Cohen’s *d* = 1.86, *P* < 0.001) (Table 2).

### Cooperative degree of anesthesia induction between the two groups

**FIGURE 3:**
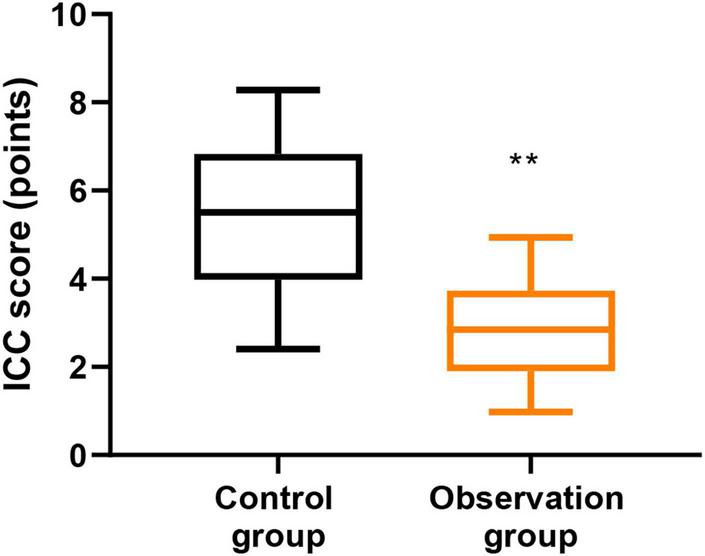
Cooperative degree of anesthesia induction between two groups. **P < 0.01.

As Figure 3 and Table 2 display, the observation group had significantly lower ICC scores than the control group (mean difference = 2.18, 95% CI [1.72, 2.64], Cohen’s *d* = 1.36, *P* < 0.001), indicating better cooperation during anesthesia induction.

### Degree of delirium at different times between the two groups

**FIGURE 4:**
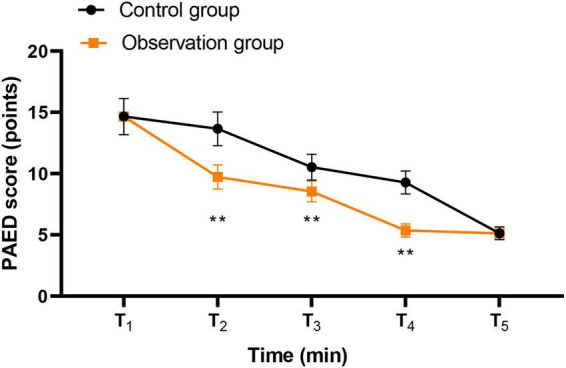
Degree of delirium at different times between two groups. **P < 0.01.

Repeated-measures ANOVA revealed a significant main effect of group (*F* = 28.36, *P* < 0.001, partial η^2^ = 0.20), a significant main effect of time (*F* = 15.72, *P* < 0.001, partial η^2^ = 0.12), and a significant group × time interaction (*F* = 9.84, *P* < 0.001, partial η^2^ = 0.08) for PAED scores. *Post hoc* comparisons showed that the observation group had significantly lower PAED scores than the control group at T_2_ (mean difference = 3.93, 95% CI [3.49, 4.37], Cohen’s *d* = 3.28, *P* < 0.001), T_3_ (mean difference = 1.98, 95% CI [1.62, 2.34], Cohen’s *d* = 2.08, *P* < 0.001), and T_4_ (mean difference = 3.91, 95% CI [3.63, 4.19], Cohen’s *d* = 5.09, P < 0.001) (Table 2). No significant differences were observed at T_1_ (*P* = 0.971) or T_5_ (*P* = 0.917), consistent with the pattern shown in Figure 4.

### Degree of pain between the two groups

**FIGURE 5:**
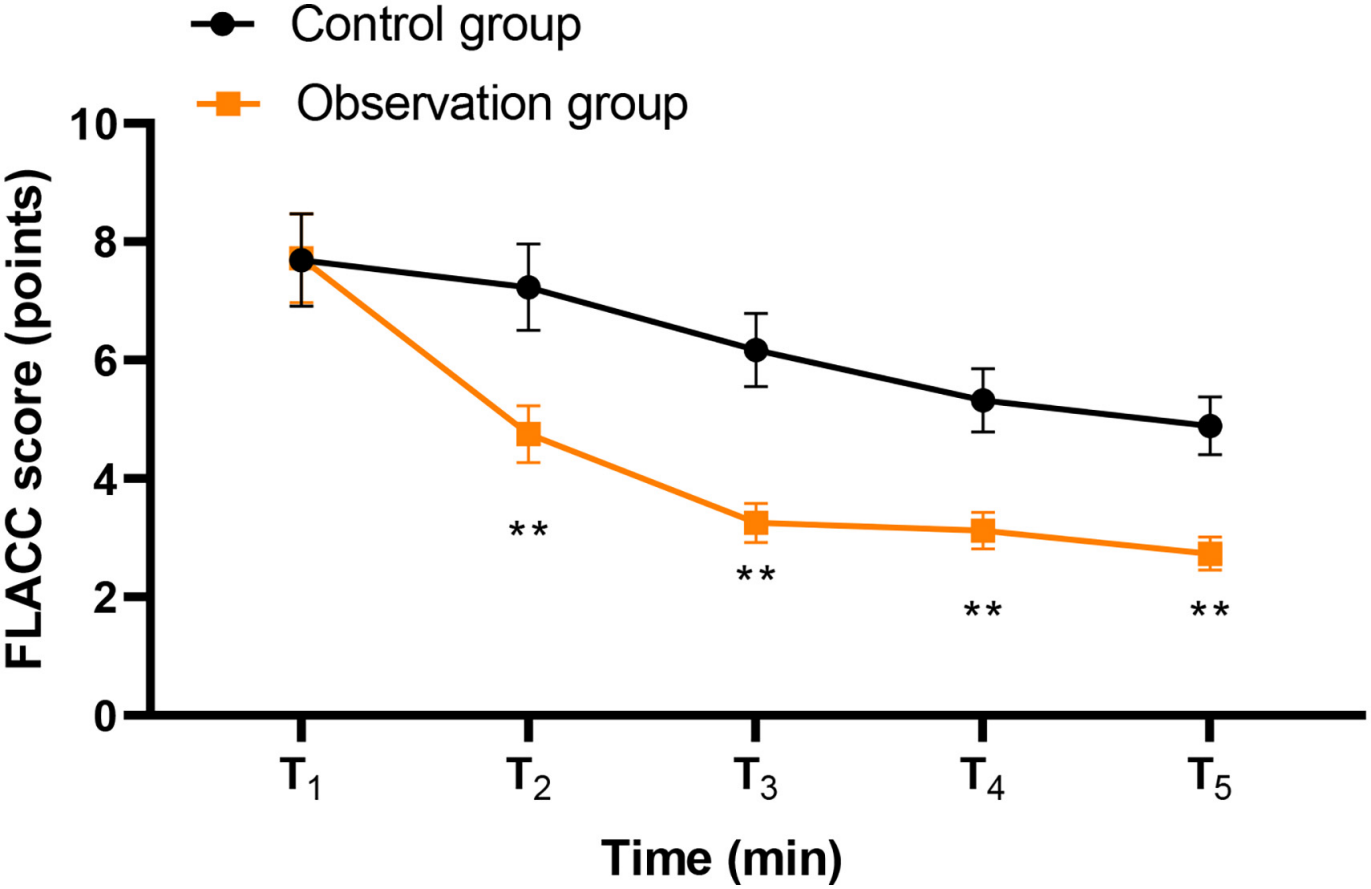
Degree of pain at different times between two groups. **P < 0.01.

Repeated-measures ANOVA for FLACC scores demonstrated a significant main effect of group (*F* = 31.52, P < 0.001, partial η^2^ = 0.22), a significant main effect of time (*F* = 22.18, *P* < 0.001, partial η^2^ = 0.16), and a significant group × time interaction (*F* = 11.36, *P* < 0.001, partial η^2^ = 0.09). *Post hoc* comparisons revealed significantly lower FLACC scores in the observation group compared with the control group at T_2_ (mean difference = 2.48, 95% CI [2.25, 2.71], Cohen’s *d* = 3.98, *P* < 0.001), T_3_ (mean difference = 2.92, 95% CI [2.74, 3.10], Cohen’s *d* = 5.85, *P* < 0.001), T_4_ (mean difference = 2.20, 95% CI [2.04, 2.36], Cohen’s *d* = 5.07, *P* < 0.001), and T_5_ (mean difference = 2.16, 95% CI [2.01, 2.31], Cohen’s *d* = 5.37, P < 0.001) (Table 2). No significant difference was observed at T_1_ (*P* = 0.832), as illustrated in Figure 5.

### Nursing satisfaction between the two groups

As Table 3 displayed, the observation group had a significantly higher total satisfaction rate than the control group (96.56% vs. 82.76%; OR = 5.83, 95% CI [1.22, 27.86], *P* = 0.014).

**TABLE 3:** Nursing satisfaction between two groups.

Groups	*n*	Very satisfied *n* (%)	Basically satisfied *n* (%)	Dissatisfied *n* (%)	Total satisfaction rate *n* (%)	OR (95% CI)	*P*-value
Control group	58	20 (34.48)	28 (48.28)	10 (17.24)	48 (82.76)	5.83 (1.22, 27.86)	0.014
Observation group	58	28 (48.28)	28 (48.28)	2 (3.44)	56 (96.56)	–	–

OR, odds ratio (observation group vs. control group for satisfaction); CI, confidence interval.

## Discussion

In the present study, parental accompanying during anesthesia induction and recovery period nursing was associated with shorter eye-opening time, shorter PACU stay, lower postoperative anxiety scores, better induction compliance, lower PAED scores at the middle PACU time points, lower FLACC scores during recovery, and higher family satisfaction. Overall, these findings suggest that a parent-accompanied perioperative nursing model may improve behavioral recovery and perioperative experience in children undergoing endoscopic plasma-assisted tonsillectomy combined with adenoidectomy. Similar directions of benefit have been reported in recent studies and meta-analyses of parental presence during induction and in the PACU, although the magnitude of effect varies across study designs and perioperative settings (3, 7, 9).

A meaningful finding in our study was that the observation group had shorter eye-opening time and a shorter stay in the anesthesia resuscitation room, whereas the recovery time of spontaneous respiration did not significantly differ between the two groups. This pattern suggests that the intervention may not have substantially altered the physiologic recovery of spontaneous breathing itself, but may instead have facilitated smoother behavioral emergence and earlier readiness for discharge from the PACU. We considered that parents are the main emotional support figures for children, and that when parents were guided to call the child’s name, encourage the child, and gently touch the child’s forehead or face during early awakening, the child’s sense of security may have been enhanced, thereby accelerating recovery. This explanation remains plausible, but an alternative interpretation should also be considered: what improved may have been the quality of emergence behavior and adaptation to the PACU environment, rather than the pharmacologic offset of anesthesia alone (7, 15).

Our anxiety-related findings also deserve careful interpretation. In the present study, the observation group showed lower mYPAS and S-TAI scores on postoperative day 1. This supports our original view that parental presence can relieve separation-related distress and reduce the child’s fear of the unfamiliar perioperative environment. At the same time, the results should not be overstated. Because anxiety was assessed before surgery and again on postoperative day 1 rather than at the exact moment of induction, the observed difference is better understood as reflecting a broader perioperative and early postoperative psychological benefit rather than a direct real-time measure of immediate peri-induction anxiety. Even so, the direction of effect is consistent with recent literature showing that parental presence during induction can reduce perioperative anxiety in children and may also improve the overall perioperative experience for families (1, 3, 16).

Moreover, compared with the control group, the observation group had lower ICC scores, lower PAED scores at T2, T3, and T4, as well as lower FLACC scores at T2, T3, T4, and T5. These findings suggest that parental accompanying during anesthesia induction and recovery-period nursing may improve children’s cooperation during anesthesia induction and may also reduce emergence delirium and pain-related behavioral distress during postoperative recovery. A plausible explanation is that when parents are guided to accompany and comfort their children during the recovery period, children may feel more secure after awakening, which may alleviate agitation and reduce hemodynamic fluctuation associated with distress. In addition, previous studies have shown that perioperative anxiety is associated with poorer postoperative pain outcomes in children. Therefore, parental accompaniment may reduce fear of the unfamiliar perioperative environment, improve emotional regulation during recovery, and thereby contribute to lower pain-related behavioral scores. This may also help explain why the observation group had a higher family satisfaction rate than the control group (17).

When interpreting the findings, it is important to consider both statistical significance and clinical relevance. For eye opening time of exhalation and stay time in the PACU, the absolute differences between groups were 1.91 and 10.69 min, respectively. Although these differences may appear modest, they carry meaningful clinical implications: shorter emergence time reduces the duration of exposure to residual anesthetics, potentially decreasing the risk of respiratory complications and facilitating earlier parent-child reunion; reduced PACU stay alleviates nursing workload and improves operating room efficiency. For outcomes such as PAED and FLACC, the large effect sizes (Cohen’s *d* > 3.0 at several time points) confirm clinically substantial reductions in emergence delirium and postoperative pain. In contrast, for outcomes without clinically meaningful differences-such as recovery time of spontaneous respiration, and PAED and FLACC scores at T_1_ and T_5_—we acknowledge that the intervention did not confer additional benefit, which is consistent with the expected pattern given that parental accompaniment was not applied during those phases. These considerations support the conclusion that the observed statistically significant differences correspond to clinically relevant improvements in key perioperative outcomes.

The present study also has several strengths. It used a randomized parallel-group design, incorporated blinded assessment of the main outcome measures, applied a relatively standardized perioperative anesthetic and nursing protocol, and evaluated multiple clinically relevant outcomes across both the induction and early postoperative recovery phases. These features strengthen the internal consistency of the study and enhance the clinical relevance of the findings. However, several limitations of this study should be acknowledged. First, the intervention described as parental accompaniment was a bundled perioperative nursing intervention that included not only parental presence during the pre-induction and early recovery phases, but also preoperative education, parental training, and distraction-based supportive strategies; therefore, the independent effect of parental presence alone could not be isolated. Second, children in the control group also received routine preoperative education, video-based counseling, and simple comforting guidance as part of standard perioperative nursing care in our institution. This may have reduced the contrast between groups, attenuated the observed between-group differences, and led to a conservative estimate of the incremental benefit of the parent-accompanied intervention. Third, no formal *a priori* sample size calculation or power analysis was performed before patient enrollment, which may have limited the statistical power of the study. Finally, anxiety was assessed before surgery and on postoperative day 1 rather than at the exact time of anesthesia induction; thus, peri-induction anxiety was not directly measured, and the anxiety-related findings should be interpreted as reflecting the broader perioperative psychological response rather than immediate induction-phase anxiety alone. Future studies with more clearly separated intervention components, larger sample sizes, and anxiety assessments conducted closer to induction are warranted.

## Conclusion

Parental accompanying during anesthesia induction and recovery period nursing was associated with lower anxiety-related scores, better induction compliance, less emergence delirium and pain-related behavioral distress, and improved recovery-room outcomes in children undergoing endoscopic plasma-assisted tonsillectomy combined with adenoidectomy. However, these findings should be interpreted cautiously in light of the bundled nature of the intervention and the methodological limitations of the study.

## Data Availability

The original contributions presented in this study are included in this article/Supplementary materials, further inquiries can be directed to the corresponding author.

## Supplementary material

Supplementary Figure 1 CONSORT flow diagram of participant enrollment, randomization, follow-up, and analysis.
