# Supplementary figures and images for: Application effect of parental accompanying during anesthesia induction and recovery period nursing in endoscopic plasma-assisted tonsillectomy combined with adenoidectomy
Source: Front Med (Lausanne). 2026 Apr 24;13:1771384. doi: 10.3389/fmed.2026.1771384 (PMC13154156; doi:10.3389/fmed.2026.1771384)

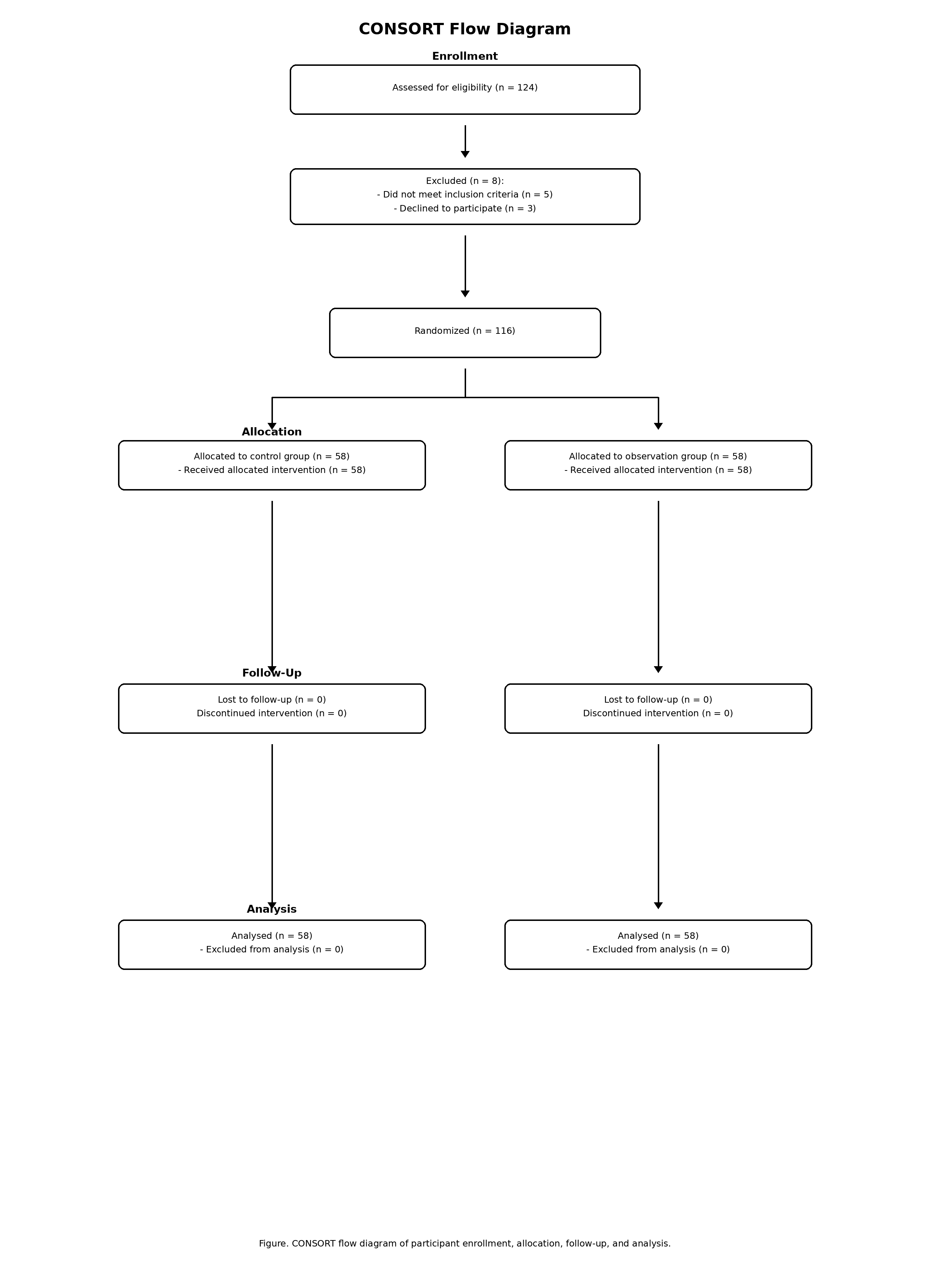

Supplement: Supplementary Figure 1 — CONSORT flow diagram of participant enrollment, randomization, follow-up, and analysis. [file Image_1.tiff]
